# A case of septicaemic anthrax in an intravenous drug user

**DOI:** 10.1186/1471-2334-11-21

**Published:** 2011-01-20

**Authors:** Arfon GMT Powell, Joseph EM Crozier, Heather Hodgson, David J Galloway

**Affiliations:** 1 Department of Surgical Gastroenterology, Gartnavel General Hospital, Glasgow, UK; 2 Tissue Viability Nurse Specialist, Gartnavel General Hospital, Glasgow, UK

## Abstract

**Background:**

In 2000, Ringertz et al described the first case of systemic anthrax caused by injecting heroin contaminated with anthrax. In 2008, there were 574 drug related deaths in Scotland, of which 336 were associated with heroin and or morphine. We report a rare case of septicaemic anthrax caused by injecting heroin contaminated with anthrax in Scotland.

**Case Presentation:**

A 32 year old intravenous drug user (IVDU), presented with a 12 hour history of increasing purulent discharge from a chronic sinus in his left groin. He had a tachycardia, pyrexia, leukocytosis and an elevated C-reactive protein (CRP). He was treated with Vancomycin, Clindamycin, Ciprofloxacin, Gentamicin and Metronidazole. Blood cultures grew *Bacillus anthracis *within 24 hours of presentation. He had a computed tomography (CT) scan and magnetic resonance imagining (MRI) of his abdomen, pelvis and thighs performed. These showed inflammatory change relating to the iliopsoas and an area of necrosis in the adductor magnus.

He underwent an exploration of his left thigh. This revealed chronically indurated subcutaneous tissues with no evidence of a collection or necrotic muscle. Treatment with Vancomycin, Ciprofloxacin and Clindamycin continued for 14 days. Negative Pressure Wound Therapy (NPWT) device was applied utilising the Venturi™ wound sealing kit. Following 4 weeks of treatment, the wound dimensions had reduced by 77%.

**Conclusions:**

Although systemic anthrax infection is rare, it should be considered when faced with severe cutaneous infection in IVDU patients. This case shows that patients with significant bacteraemia may present with no signs of haemodynamic compromise. Prompt recognition and treatment with high dose IV antimicrobial therapy increases the likelihood of survival. The use of simple wound therapy adjuncts such as NPWT can give excellent wound healing results.

## Background

Anthrax is believed to be the cause of the 5^th ^plague described in the book of Exodus (chap 9:3). It is a zoonosis affecting most herbivores, however, transmission from human to human has never been documented [1-3]. It is caused by *Bacillus anthracis*, a gram positive spore forming bacillus. It occurs when *Bacillus anthracis*' endospores enter the body either through breaks in the skin, ingestion or inhalation. Anthrax characterisation is based upon its original mode of transmission; cutaneous, gastrointestinal and inhalational. Anthrax is derived from the Greek word meaning coal after the black skin lesions seen in its cutaneous form [1].

Anthrax remains relatively rare with between 20,000 and 100,000 cases occurring in the world annually [4]. It is predominantly related to occupational exposure as seen in farmers, veterinarians and people handling wool [5]. Only a handful of anthrax cases have been seen in Britain over the last decade. In 2006, a fatal case of inhalation anthrax was reported in the Scottish borders [6]. This was the first case of anthrax seen in nearly 30 years. In 2000, Ringertz et al described the first case of systemic anthrax caused by injecting heroin contaminated with anthrax [7].

In 2008, there were 574 drug related deaths in Scotland, of which 336 were associated with heroin and or morphine [8]. Of the 574 deaths, 197 (34%) occurred in Greater Glasgow & Clyde. NHS Greater Glasgow and Clyde covers the city of Glasgow and a population of approximately (1.2 million) [9]. In 2000, it was estimated that 6809 people in Glasgow were intravenous drug users (IVDU) [10]. Due to the risky behaviour associated with illicit drug use, mortality rates in IVDU patients have been estimated to be 12-22% higher than an age-adjusted population [11-13].

In December 2009, the first case of fatal anthrax relating to intravenous drug abuse was documented in the British media [14]. In total there were 47 confirmed cases and 13 anthrax related deaths during this outbreak [15,16]. Systemic anthrax infection is associated with high mortality rates with 45% of patients with inhalation anthrax ultimately dying from the infection [17,18]. Cutaneous anthrax infection, however, is not associated with high mortality rates [19].

We present an interesting case of septicaemic anthrax, in a patient with no evidence of septic shock, caused by injecting heroin contaminated with anthrax in the UK. In our case report, we discuss the clinical and pathological aspects of the case, the use of wound therapy adjuncts in promoting wound healing and review the current literature.

## Case Presentation

A 32 year old male who was a known IVDU, presented with a 12 hour history of increasing swelling in his left leg. He had a purulent discharge from a chronic sinus in his left groin.

**Figure 1:**
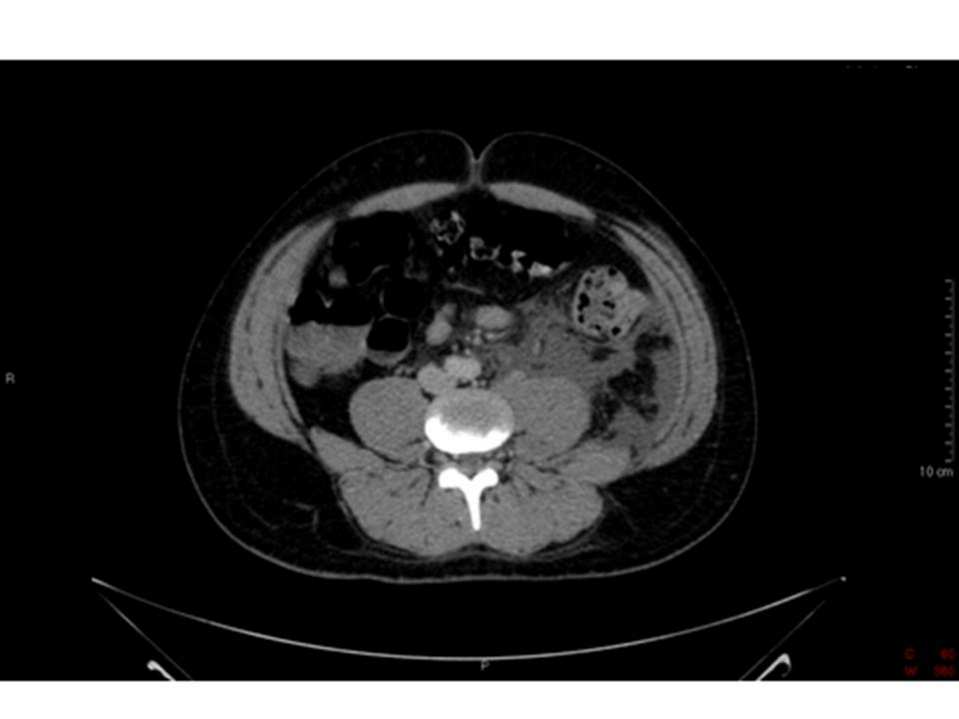
**CT Abdomen and Pelvis**. Loculated fluid and inflammatory change anterior to the left psoas muscle.

**Figure 2:**
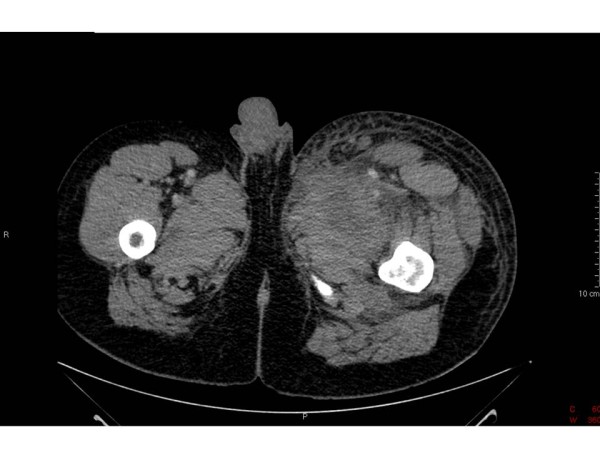
**CT Abdomen and Pelvis**. The adductor magnus which is oedematous possibly reflecting muscle necrosis.

On examination, he had warm erythematous sinuses in both groins. The left sinus was discharging foul smelling pus. He had a tachycardia 120 bpm and pyrexia 38.2 degrees C, blood pressure (BP) 136/86. He was alert and orientated to time, place and person. Blood tests revealed a white cell count (WCC) of 15.9 × 10^9^/l, a C-Reactive Protein (CRP) of 21 mg/dl and blood cultures were taken. He was treated with broad spectrum antibiotics Vancomycin, Clindamycin, Ciprofloxacin, Gentamicin and Metronidazole in line with the latest advice from microbiology because of a recent outbreak of anthrax in the local IVDU population. Within 24 hours of collection, all 6 blood culture bottles grew gram positive organisms suggestive of *Bacillus anthracis *and he was referred for consideration of surgery. The cultured *Bacillus anthracis *organism was susceptible to all the above antibiotics. Clinically there was no abscess to drain so he had a computed tomography (CT) scan of his abdomen, pelvis and thighs performed. This showed loculated fluid and inflammatory change anterior to the left psoas muscle extending down to the iliacus [Figure 1]. In the pelvis, the left pectineus and adductor magnus were oedematous possibly reflecting muscle necrosis [Figure 2].

**Figure 3:**
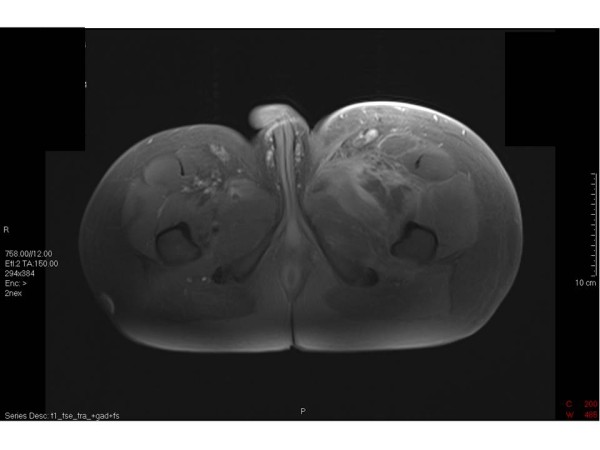
**MRI Pelvis and Thighs**. An area of decreased signal inferior and lateral to the left femoral neurovascular bundle. This was suggestive of tissue necrosis.

Given that he was clinically well his conservative management and monitoring continued. Over the next five days there were signs of improvement with a reduction in the inflammatory markers and no evidence of organ dysfunction. In consultation with colleagues in microbiology, infectious diseases and public health a further CT scan was performed to ensure that there was no collection to drain as the limited local experience suggested that drainage and debridement of collections and necrotic tissues resulted in optimal management. This scan showed that the retroperitoneal and thigh changes had improved slightly. Within a further 24 hours he had developed an area of cellulitis over the left thigh and lower abdomen and a Magnetic Resonance Imaging (MRI) was carried out. This showed diffuse oedematous change in the muscles and subcutaneous fat of the left leg from the level of the inguinal region to below the knee. After contrast, there were areas which did not show evidence of contrast enhancement with an irregular enhancing margin. The absence of discrete high signal on the STIR images was thought to represent areas of non-perfused musculature, possible undergoing necrotic change [Figure 3].

He underwent an exploration of his left thigh. This revealed chronically indurated subcutaneous tissues in keeping with his long history of injecting into the area. There was some oedematous fluid but no evidence of a collection or necrotic muscle. Post-operatively he made a steady recovery.

Treatment with Vancomycin, Ciprofloxacin and Clindamycin continued for 14 days. Negative Pressure Wound Therapy (NPWT) device was applied utilising the Venturi™ wound sealing kit. This followed the therapy application technique first described by Chariker et al using saline-moistened gauze, a silicone drain and clear, semi-permeable adhesive film, together with lower pressures, usually between 60-80 mmHg.^15^

**Figure 4:**
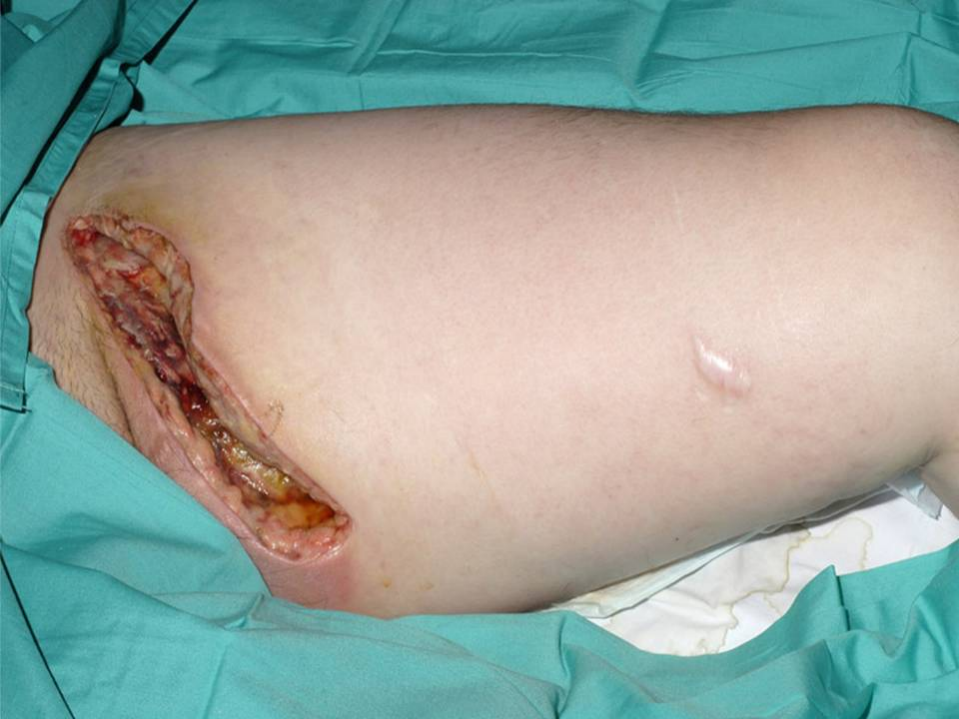
**Appearance of the wound prior to the use of NPWT**.

**Figure 5:**
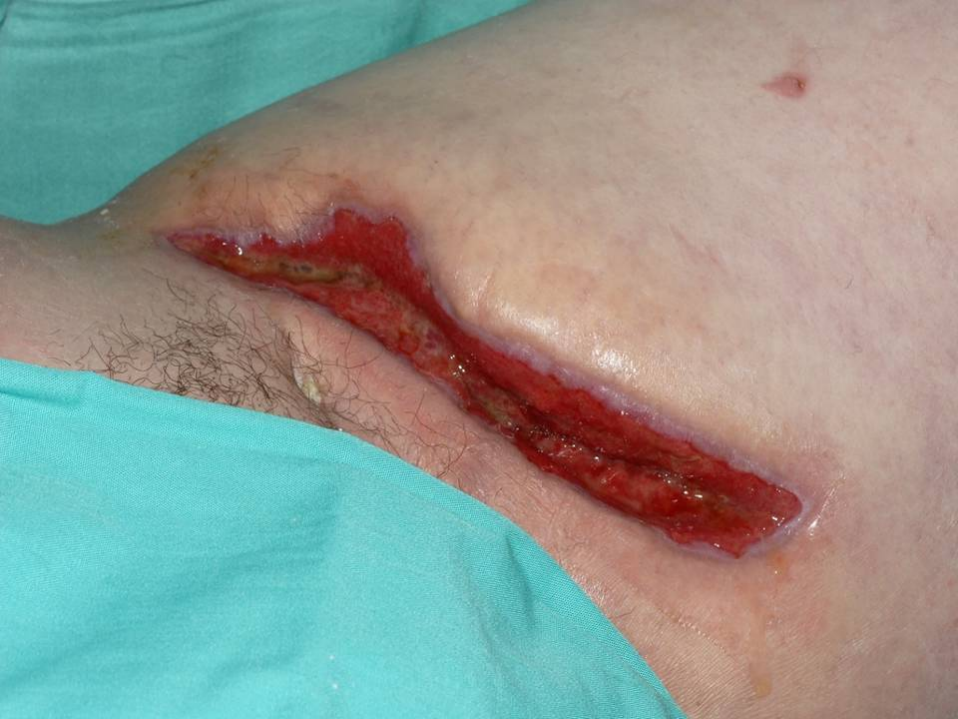
**Appearance of the wound following 4 weeks of NPWT**.

The dressings were changed three times per week and wound exudate collection canisters changed as required when full. He was discharged on a 4 week course of oral Ciprofloxacin 400 mg twice a day and continued with NPWT. Following 4 weeks of treatment, the wound dimensions had reduced from 300 cm3 [Figure 4] to 68 cm3 [Figure 5], a reduction in size of 77%. He remains well and attends regular review at outpatient clinic.

## Conclusions

We present an interesting case of septicaemic anthrax in an intravenous drug user, who despite having *B. anthracis *bacteraemia and severe cutaneous infection, displayed no evidence of septic shock and made an uneventful recovery following IV antibiotic therapy and surgical debridement. This case was part of a large outbreak of septicaemic anthrax infection in an IVDU population of 47 patients with a mortality rate of 27% [15].

Mortality associated with systemic anthrax infection is well known to be high with 40% of the patients in the 2001 bioterrorist attacks dying from the infection [17]. Of the 10 patients in their series, 7 patients presented with severe sepsis and grew *B. anthracis *on blood culture. Of the 7 patients with bacteraemia 4 died of acute circulatory collapse. This suggests the presence of positive blood cultures foretells a high risk of mortality. Doganay and colleagues described a series of 22 patients presenting with cutaneous anthrax infection over a 7 year period [19]. Of these, 10 patients had severe infection and 2 patients suffered from septic shock. In contrast to our case, all patients with positive blood culture had evidence of severe infection and septic shock. Of interest, none of the patients in their series died from anthrax.

It is believed this outbreak resulted from exposure to a batch of contaminated heroin. This is suggested by the geographical distribution and the occurrence of contemporaneous outbreaks within the city. McGuigan et al reported a similar outbreak in 2000; however, the aetiological agent is thought to have been *Clostridium novyi *[13]. Street heroin in Britain is thought to originate from Afghanistan [13,20]. Non-sterile methods for its production, 'cutting' and transportation increase the risk of contamination.

The lethal outbreak of *Clostridium novyi *in 2000 prompted the production of consensus guidelines for treating severe cutaneous infection in IVDU patients. In the case series reported by McGuigan et al, the commonest organisms seen, were *Clostridium novyi*, *Staphylococcus aureus*, group A beta-haemolytic streptococci and anaerobes. Based on this finding they suggested using an antimicrobial regimen of Flucloxacillin, Benzylpenicillin, Gentamicin, Clindamycin and Metronidazole [13].

In 2001, Swartz published guidelines for the recognition and management of anthrax. He suggested using ciprofloxacin 400 mg IV combined with a penicillin for inhalation and severe cutaneous anthrax infection [21]. His recommendations were based on consensus guidelines published by Iglesby et al. The efficacy of ciprofloxacin in anthrax had been poorly studied in humans, however, in animal models excellent recovery has been demonstrated despite the lack of an immune response [22]. This finding led to the recommendation that all patients surviving anthrax should continue antibiotic therapy for 60 days.

The evidence published by McGuigan et al suggested that of all the patients who were classified as a 'definite' diagnosis of *Clostridium novyi*, approximately 20% grew an alternative organism. This suggests, even during a recognised outbreak, the diagnosis of a particular infection based on clinical parameters alone is unreliable.

All IVDU patients presenting with severe cutaneous infection were treated with Benzylpenicillin, Flucloxacillin, Gentamicin, Clindamycin, Ciprofloxacin and Metronidazole. In the case of penicillin allergy Vancomycin was used in place of Benzylpenicillin and Flucloxacillin. Once a causative agent was found, the antimicrobial therapy was rationalised. The diagnosis of anthrax was confirmed by isolation of *Bacillus anthracis *in early blood cultures in some patients. This was supported by PCR testing of blood or excised tissues at the Health Protection Agency (HPA) Special Pathogens Reference Unit (SPRU) at Porton Down.

NPWT is a non-invasive technology comprising a negative pressure pump connected by a tube to a dressing that occupies the wound cavity. The dressing is sealed to the peri-wound skin using an adhesive film. This provides a closed system, so that negative (sub-atmospheric) pressure is generated at the wound/dressing interface. NPWT is suitable for acute, chronic and traumatic wounds as an adjunct to surgery [23]. Despite NPWT appearing in the literature for over 50 years, the physiological and molecular biological mechanisms by which NPWT accelerates wound healing remain largely unknown [24].

Although systemic anthrax infection is rare, it should be considered when faced with severe cutaneous infection in IVDU patients. The presence of *Bacillus anthracis *on blood culture is normally associated with septic shock and high rates of mortality; however, our case suggests that these patients may not exhibit haemodynamic compromise despite having significant bacteraemia. Prompt recognition and treatment with high dose IV antimicrobial therapy increases the likelihood of survival. Adherence to antimicrobial prescribing guidelines reduces the possibility of missing other potentially lethal organisms. The use of simple wound therapy adjuncts such NPWT can give excellent wound healing results over a relatively short period.

## Consent

Written informed consent was obtained from the patient for publication of this case report and any accompanying images. A copy of the written consent is available for review by the Editor-in-Chief of this journal.

## Competing interests

The authors declare that they have no competing interests.

## Authors' contributions

AGMTP carried out the literature review, prepared the figures and drafted the manuscript. JEMC coordinated and helped draft the manuscript. HH supplied the wound images and helped draft the manuscript. DJG coordination and helped draft the manuscript. All authors read and approved the final manuscript.

## Pre-publication history

The pre-publication history for this paper can be accessed here:

http://www.biomedcentral.com/1471-2334/11/21/prepub
